# Colocalization of Mating-Induced Fos and D2-Like Dopamine Receptors in the Medial Preoptic Area: Influence of Sexual Experience

**DOI:** 10.3389/fnbeh.2016.00075

**Published:** 2016-04-18

**Authors:** Victoria L. Nutsch, Ryan G. Will, Christopher L. Robison, Julia R. Martz, Daniel J. Tobiansky, Juan M. Dominguez

**Affiliations:** ^1^Institute for Neuroscience, The University of Texas at AustinAustin, TX, USA; ^2^Department of Psychology, The University of Texas at AustinAustin, TX, USA

**Keywords:** copulation, dopamine, D2 dopamine receptors, sexual experience, preoptic area, Fos

## Abstract

Dopamine in the medial preoptic area (mPOA) stimulates sexual activity in males. This is evidenced by microdialysis and microinjection experiments revealing that dopamine receptor antagonists in the mPOA inhibit sexual activity, whereas agonists facilitate behavior. Microdialysis experiments similarly show a facilitative role for dopamine, as levels of dopamine in the mPOA increase with mating. While the majority of evidence suggests an important role for dopamine receptors in the mPOA in the regulation of male sexual behaviors, whether sexual activity or sexual experience influence dopamine receptor function in the mPOA has not been previously shown. Here we used immunohistochemical assays to determine whether varying levels of sexual activity or experience influence the number of cells containing Fos or D2 receptor immunoreactivity. Results show that sexual experience facilitated subsequent behavior, namely experience decreased latencies. Moreover, the number of cells with immunoreactivity for Fos or D2 correlated with levels of sexual experience and sexual activity. Sexual activity increased Fos immunoreactivity. Sexually experienced animals also had significantly more D2-positive cells. Sexually inexperienced animals copulating for the first time had a larger percentage of D2-positive cells containing Fos, when compared to sexually experienced animals. Finally, regardless of experience, animals that had sex prior to sacrifice had significantly more D2-positive cells that contained Fos, vs. animals that did not copulate. These findings are noteworthy because sexually experienced animals display increased sexual efficiency. The differences in activation of D2 and changes in receptor density may play a role in this efficiency and other behavioral changes across sexual experience.

## Introduction

Dopamine plays an important role in the regulation of male sexual behavior (Hull and Dominguez, 2015). Consider, for example, earlier reports showing that systemic administration of L-DOPA results in a greater number of rats displaying sexual activity (Da Prada et al., 1973; Paglietti et al., 1978). Administration of apomorphine, a D1/D2 dopamine receptor agonist, also results in rats requiring less time to reach an ejaculation and a larger percent of them achieving ejaculations (Paglietti et al., 1978). In addition to enhancing the consummatory aspects of behavior, dopamine agonists boost psychogenic erections and sexual motivation. Animals receiving the selective D2/D3 DA receptor agonists, 7-OH-DPAT or B-HT 920, displayed a greater number of psychogenic erections in the presence of an inaccessible estrous female when compared with rats receiving vehicle control (Ferrari et al., 2002). While agonists facilitate sexual behavior, antagonists appear to impair behavior. For instance, administration of the D2-receptor antagonist haloperidol decreases the number of mounts, intromissions, and ejaculations, and increases mount and intromission latencies in rats (Pfaus and Phillips, 1989). Administration of dopamine antagonists also inhibit anticipatory sexual behavior, as sexually experienced male rats receiving drugs display fewer anticipatory level changes, before the introduction of a sexually receptive female (Pfaus and Phillips, 1991) in a bilevel chamber that is used as an assay of sexual motivation (Mendelson and Pfaus, 1989). Finally, it appears that central, not peripheral, dopamine receptors facilitate erectile response, since erections elicited by systemically administered apomorphine were blocked by haloperidol (a centrally active dopamine antagonist) but not domperidone (a peripherally active dopamine antagonist) in mice (Rampin et al., 2003) and in rats (Pehek et al., 1988a).

While several brain areas orchestrate to regulate sexual behaviors, one region in particular, the medial preoptic area (mPOA) is the most extensively studied brain region relating to the regulation of male sexual behavior in all vertebrate species (Hull and Dominguez, 2015). This emphasis is justifiable, as the mPOA receives indirect input from every sensory modality (Simerly and Swanson, 1986) and sends projections to structures that are critical for the initiation and patterning of copulation (Simerly and Swanson, 1988). A number of neurotransmitters and hormones influence sexual function by acting in this area (Hull et al., 1994; Dominguez and Hull, 2005; Dominguez et al., 2006; Hull and Dominguez, 2006; Dominguez, 2009; Kleitz-Nelson et al., 2010a,b). Ablation studies confirm a role for this region in the regulation of sexual behaviors; lesions here significantly impair several aspects of behavior (Hull and Dominguez, 2015). Conversely, electrical stimulation of the mPOA in rats reduced the number of intromissions required for ejaculation, the time preceding an ejaculation, and the postejaculatory interval (Malsbury, 1971; Rodríguez-Manzo et al., 2000). Stimulation of the mPOA also elicits erections and the urethrogenital reflex, a model for orgasm (Marson and McKenna, 1994; Giuliano et al., 1997).

Not surprisingly, sexual activity also increases neural activity in the mPOA. For instance, electrophysiological recordings in the mPOA of sexually experienced monkeys showed increased activity both when the animals lever-pressed to bring a conspecific female closer and also during copulation, while activity ceased after ejaculation (Oomura et al., 1988). Other experiments, using similar electrophysiological recordings in rats also showed that mating induces increased activity in the mPOA (Shimura et al., 1994). Immunohistochemical data mirror the electrophysiological results. Fos-immunoreactivity (ir), used as a measure of cellular activity, increases in the mPOA after males are exposed to the odor of an estrous female (Bressler and Baum, 1996; Pfaus and Heeb, 1997; Tobiansky et al., 2012). When comparing Fos-ir in the mPOA of copulating animals, vs. controls, increasing amounts of copulation induce increasing amounts of Fos-ir in the mPOA of male rats (Robertson et al., 1991; Baum and Everitt, 1992; Veening and Coolen, 1998; Nutsch et al., 2014).

Whether the mPOA also modulates behavioral changes that result from sexual experience is still not entirely clear. However, several studies suggest that the mPOA is at least partly involved in these experience-induced changes. Consider, for example, the following studies that linked sexual experience with changes in the mPOA: sexual experience influences mating-induced activity in nitric oxide synthase-containing cells in the mPOA of male rats (Nutsch et al., 2014); the number of astrocytes in the mPOA negatively correlated with latency to ejaculate in sexually inexperienced but not experienced male rats (Will et al., 2015); sexual experience increases oxytocin receptor protein and gene expression in the mPOA of male rats (Gil et al., 2011); experience-induced enhancement of male sexual behavior involves dopamine D1 receptors and phosphorylation of dopamine- and cyclic-AMP-regulated phosphoprotein-32 in the mPOA of male rats (McHenry et al., 2012); sexual experience increased androgen receptors in the mPOA of male mice (Swaney et al., 2012); sexual experience increased nitric oxide synthase in the mPOA of male rats. Here we focus on mating induced stimulation of D2-receptor containing cells, and whether this stimulation is influenced by sexual experience.

Given the mPOA’s integrative and central role in the regulation of male sexual behavior, it may not be surprising that dopamine facilitates sexual activity by acting in this region (Dominguez and Hull, 2005). Sources of dopamine to the mPOA include dopamine-producing cells in the periventricular nucleus (A14; Moore and Lookingland, 1995), the rostral zona incerta (A13; Björklund et al., 1975), and (at least in female rats) also the ventral tegmental area (A10; Miller and Lonstein, 2009). Studies employing selective lesions, microinjections, or microdialysis techniques back a close link between dopamine activity in the mPOA and increased sexual function in males. Consider that 6-OHDA lesions of dopamine fibers in the mPOA, 1 week before testing, combined with acute depletion of dopamine synthesis in A14 resulted in fewer ejaculations, longer ejaculation latencies and longer post-ejaculatory intervals (PEI; Bitran et al., 1988). Microinjections of dopamine antagonists have an equally deleterious effect. Microinjections of cis-flupenthixol into the mPOA result in fewer rats copulating, and those that copulate achieve fewer ejaculations (Pehek et al., 1988b). Microinjections of dopamine antagonists also impair penile reflexes, specifically microinjections of cis-flupenthixol decreased ex copula penile reflexes. Conversely, microinjections of dopamine agonists enhance behavior. Apomorphine microinjections increase the number of ejaculations and decrease the time required to achieve an ejaculation and the time spent in PEI (Hull et al., 1986). Apomorphine microinjections into the mPOA also decrease latency to the first penile reflex and increase the number of erections in a timed test (Pehek et al., 1989), showing an enhanced penile response following dopamine receptor stimulation in the mPOA.

Lastly, microdialysis experiments show increased release of dopamine in the mPOA of rats following precopulatory exposure to an estrous female and during copulation (Hull and Dominguez, 2015). Further evidence that dopamine in the mPOA contributes to sexual motivation, not merely general arousal, was provided by Kleitz-Nelson et al. (2010a,b) using Japanese quail, which exhibit a shorter temporal pattern of copulation than rats and do not have an intromittent organ. These studies showed that levels of dopamine increased in the presence of a female, returning to baseline after removal of the female; however, quails that failed to copulate did not display this increased release (Kleitz-Nelson et al., 2010b). Conversely, males that showed a substantial increase in dopamine during precopulatory interactions behind a barrier readily copulated with females after its removal (Kleitz-Nelson et al., 2010a).

While the preponderance of evidence supports an important regulatory role for dopamine and its receptors in the mPOA, whether varying levels of sexual activity or sexual experience differentially stimulate dopamine-receptor containing cells in the mPOA was hitherto unknown. To this end, we employed immunohistochemical assays to determine whether varying levels of sexual activity or experience influence the number of cells containing D2-like dopamine receptors, Fos, or both in the mPOA of male rats.

## Materials and Methods

### Subjects

Sixty Long–Evans male rats (Harlan, Indianapolis, IN; 90 days old at arrival) were housed individually in large plastic cages, in a climate-controlled room, on a 14:10 h light/dark cycle, with lights off at 10:00 a.m. and/on at 8:00 p.m. Food and water were freely available. Conspecific females (*n* = 17) were ovariectomized under ketamine hydrochloride (50 mg/kg) and xylazine hydrochloride (4 mg/kg) anesthesia. They were brought into behavioral estrus with 4 μg estradiol benzoate (s.c.) 48 h before, and 400 μg (s.c.) progesterone 4 h before testing. Behavioral receptivity was confirmed by placing the female with a stud male shortly before the test began. All procedures were done in accordance with the National Institutes of Health Guidelines for the Use of Animals and were approved by the Institutional Animal Care and Use Committee at the University of Texas at Austin.

Male rats were randomly assigned to one of the following four conditions: animals that were sexually experienced, but did not mate on the day of sacrifice (experienced but no sex, Exp-NoSex); animals who were sexually experienced and also mated on the day of sacrifice (experienced and sex, Exp-Sex); animals who were sexually naïve and did not mate on the day of sacrifice (inexperienced and no sex, Inexp-NoSex); animals who were sexually naïve but experienced mating for the first time on the day of sacrifice (inexperienced and sex, Inexp-Sex).

Sexual experience consisted of mating with a sexually receptive female for 90 min, every other day, for 6 days before the day of sacrifice, for a total of 9 h. On the 6th day, animals were observed to confirm that they achieved at least two ejaculations during the final experience session. Three animals that did not meet this criterion were excluded from further testing. Two days separated the last experience day and the test day, when animals were sacrificed. Behavioral data were obtained and analyzed on the test day, which was 2 days after the final experience session. Animals in the mated groups were allowed to copulate to one ejaculation. Animals that failed to copulate after 1 h were removed and excluded from further analysis. No-sex controls were handled, but females were not introduced into their home cage. All animals were sacrificed with an overdose of sodium pentobarbital (100 mg/kg), 1 h after ejaculation or the end of testing.

### Immunohistochemistry

Rats were perfused transcardially with saline under pentobarbital anesthesia, followed by 4% paraformaldehyde in 0.1 M phosphate buffer (PB; pH = 7.35). Brains were removed, postfixed for 1 h in the same fixative at room temperature, and stored in 30% sucrose at 4°C. Coronal sections were cut at 35 μm and stored in cryoprotectant solution. Sections containing the mPOA underwent immunohistochemical staining for Fos and D2 dopamine receptors. Washes in PB, 4× for 5 min, preceded all incubations. Sections underwent the following incubations: 1% H_2_O_2_ in PB, and then blocked in 2% normal goat serum and 1% Tween-20 (blocking solution); mouse anti Fos primary antibody (1:5000; Santa Cruz Biotechnology, Santa Cruz, CA, USA) in blocking solution, overnight at room temperature. The following day, sections were incubated in anti-mouse biotinylated secondary antibody (1:500 in blocking solution; Vector Labs, Burlingame, CA, USA). Immunoreactivity was visualized with a diaminobenzidine (DAB)–nickel chromogen solution (Sigma, St. Louis, MO, USA) to yield a purple-black precipitate, incubation lasted 10 min. After washing thoroughly with PB, sections were then incubated with rabbit anti-D2 primary antibody and blocking solution, overnight at room temperature (1:6000; EMD Millipore, Billerica, MA, USA). The following day, incubation in anti-rabbit biotinylated secondary antibody (1:500 in blocking solution; Vector Labs, Burlingame, CA, USA) preceded the avidin-biotin conjugate, and was visualized with a DAB chromogen solution without nickel, yielding a brown precipitate. Sections were dehydrated, mounted, and coverslipped with DPX (VWR, Radnor, PA, USA). For negative controls, sections underwent the same immunostaining procedure, except the D2, Fos, or both primary antibodies were excluded. When introducing and removing tissue from incubations, experimenters were careful to minimize the transfer time separating the first and last set of tissue, the transfer time averaged 40 s for all incubations including DAB.

Light microscopy was used to quantify the number of cells containing D2-ir, Fos-ir, and double-labeled cells. The mPOA was examined bilaterally and immunolabeled cells were counted in a 300 × 400 μm area in the medial preoptic nucleus (MPN), a central nucleus in the mPOA. Counts were performed manually using ImageJ. Brains were sliced at 35 μm into four equal sections. Tissue was analyzed from one of these sections, thus there was a 105 μm separating each slice. Cell counts were averaged across both hemispheres and across the six sections. Six sections for each animal were counted bilaterally, according to coordinates from Swanson (2004).

### Western Immunoblotting

For immunohistochemistry, we stained using a rabbit polyclonal anti-D2 dopamine receptor. The manufacturer’s description of the D2 antibody states that it recognizes the D2 receptor in rats, does not cross-react with other dopamine receptors, and exhibits minimal cross-reactivity with the short-form (D2Sh) of the receptor. This is important to note because, while the D2Sh is situated primarily pre-synaptically (viz. autoreceptor), the D2Lh long-form functions more as a classical post-synaptic receptor, and our quantifications were of D2-containing cells not fibers. Additionally, the predicted size of D2 is approximately 50 kDa. However, the manufacturer’s own immunoblotting experiments using this D2 antibody detected two bands at ~48 and ~51 kDa. Immunoblotting experiments using other D2 antibodies also report bands beyond 50 kDa (Farooqui et al., 1992; Sakata et al., 1992; Boundy et al., 1993). For this reason, we performed Western immunoblotting experiments to test the specificity of the D2 antibody.

**Figure 1:**
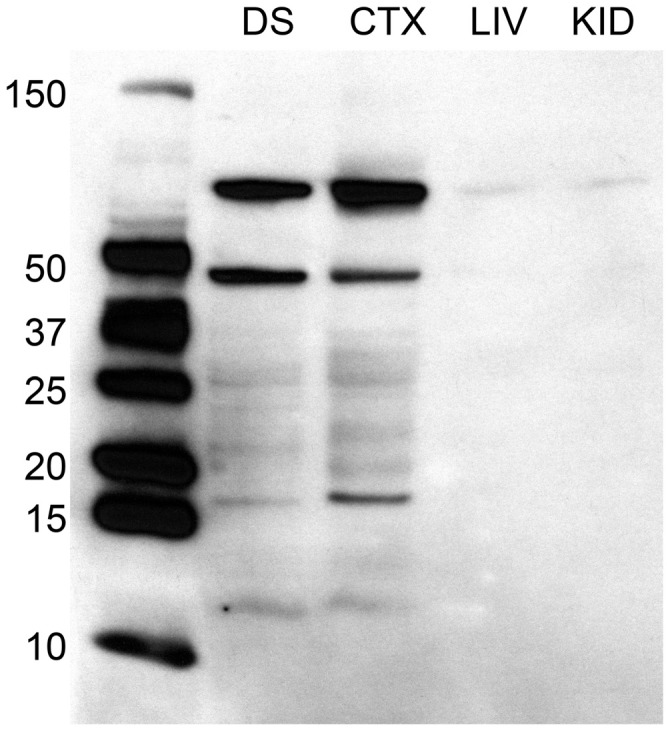
**Western immunoblotting analysis of antibody against D2 dopamine receptor protein, raised in rabbit.** The ladder depicting molecular weight is on the left of the dorsal striatum (DS), posterior cortex (CTX), liver (LIV), and kidney (KID) samples to the right. Molecular weight of the D2 receptor protein is 51 kDa. Immunoblotting analyses reveals a band at ~50 kDa, in addition to other bands, in the brain samples. This band is absent in both liver and kidney samples.

For Western immunoblotting, brain samples were homogenized and purified in RIPA buffer (Pierce) with protease inhibitor tablets (Roche), and protein content was estimated using a NanoDrop system. 10 μg protein load volume was separated by electrophoresis and compared using PrecisionPlus unstained standards (BioRad). As positive controls, brain samples were collected from regions known to have relatively high concentration of D2 receptors, namely the dorsal striatum (DS) and posterior cortex (CTX; Lidow et al., 1989; Meador-Woodruff et al., 1989); negative controls included samples collected from the liver (LIV) and kidney (KID). Samples were transferred to PVDF and exposed to rabbit anti-D2 antibody (1:4000, Millipore) and then goat anti-rabbit HRP secondary (1:30,000, Bio-Rad) in a blocking buffer containing 2% normal goat serum. Bands were visualized using ECL chemiluminescence. Results confirmed the presence of a band at ~50 kDa, however we found additional bands beyond 50 kDa (see Figure 1). Henceforth we designate D2 immuno-positive staining in our experiments as indicative of putative D2-like dopamine receptors.

### Data Analysis

A two-way analysis of variance (ANOVA; mating × experience) was performed to probe for differences in the number of immuno-positive cells. Also, Welch two sample *t*-tests were used to probe for differences in behavioral measures. Data analyses were performed with R (version 3.2.2).

## Results

**Figure 2:**
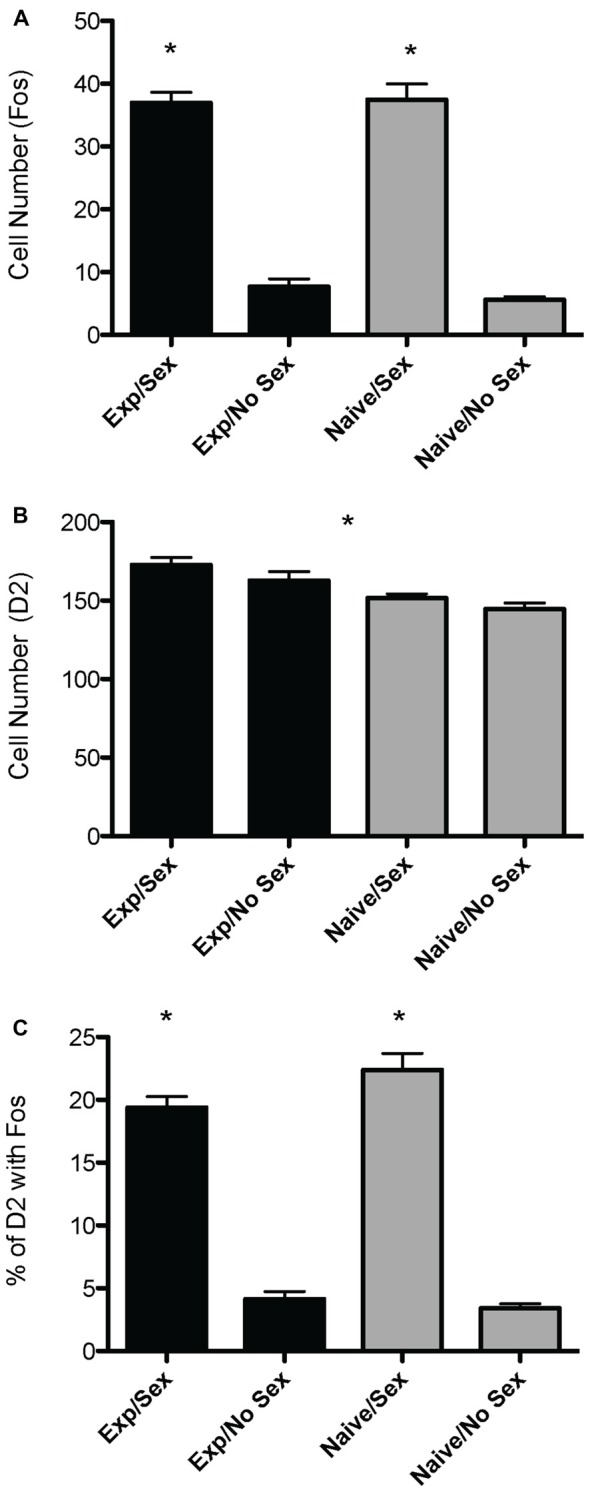
**Effects of sexual activity and sexual experience on D2- and Fos-immunoreactivity in the mPOA of male rats. (A)** Animals who copulated prior to sacrifice had significantly more Fos-positive cells. **(B)** Animals with sexual experience had significantly more D2-immunopositive cells compared to naïve animals. **(C)** There was an interaction between sex and experience on the percentage of D2 cells that contained Fos; specifically, naïve animals copulating for the first time had a significantly higher percent of D2 cells that expressed Fos. (*p < 0.05).

**Figure 3:**
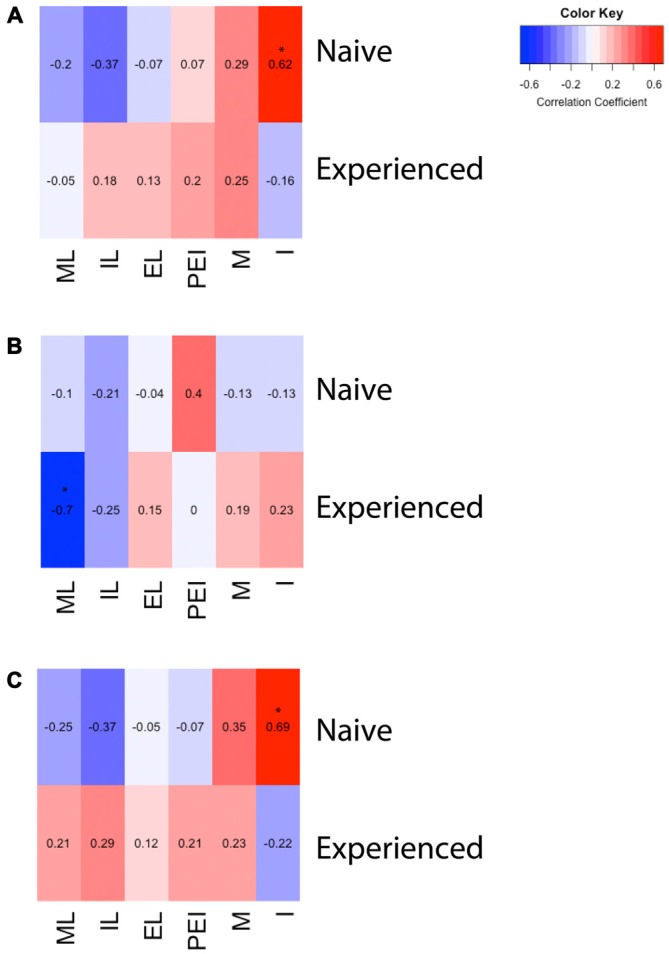
**Heat maps depicting correlation between sexual behavior and D2- or Fos-immunoreactivity in the mPOA of male rats. (A)** There was a significant positive correlation between the number of intromissions and Fos immunoreactivity in naïve animals copulating for the first time. **(B)** There was a significant negative correlation between latency to mount and the number of D2 immunopositive cells in sexually experienced animals. **(C)** There was a significant positive correlation between the number of intromissions and percent of D2 cells that contained Fos in naïve animals copulating for the first time. (ML, mount latency; IL, intromission latency; EL, ejaculation latency; PEI, post ejaculation interval; M, mounts; I, intromissions).

A two way ANOVA revealed a significant main effect of sex on the number of Fos-positive cells in the mPOA (*F*_(1,40)_ = 178.915, *p* < 0.001), whereby animals that had sex prior to sacrifice had significantly more Fos-ir cells. However, there was not a main effect of experience (*F*_(1,40)_ = 0.800, *p* = 0.376), nor was there a sex by experience interaction (*F*_(1,40)_ = 0.562, *p* = 0.4579; Figure 2A). A heat map depicting the relationship between the number of Fos-positive cells and sexual behavior is presented in Figure 3A.

A two way ANOVA revealed a significant main effect of experience on the number of putative D2-ir cells in the mPOA (*F*_(1,40)_ = 7.417, *p* < 0.01) whereby sexually experienced animals had significantly more D2-ir cells regardless of sexual activity prior to sacrifice (experienced, 167 ± 3.9; inexperienced 148 ± 2.3; see Figure 2B). However, there was not a main effect of sex (*F*_(1,40)_ = 2.564, *p* = 0.117), nor was there a sex by experience interaction (*F*_(1,40)_ = 0.119, *p* = 0.732; Figure 3B). A heat map depicting the relationship between the amount of D2-ir cells and sexual behavior is presented in Figure 3B.

A two-way ANOVA revealed a significant main effect of sex on the percent of D2-ir cells in the mPOA that expressed Fos (*F*_(1,40)_ = 162.831, *p* < 0.001), but not a main effect of experience (*F*_(1,40)_ = 0.324, *p* = 0.572). However, there was a significant sex by experience interaction (*F*_(1,40)_ = 4.390, *p* < 0.05). Decomposition of this significant interaction revealed sexually naïve animals copulating for the first time had a significantly higher fraction of D2-positive cells that expressed Fos compared to sexually experienced animals, see Figure 2C. However, there was not a significant difference in the fraction of D2-positive cells that expressed Fos between sexually naïve and experienced animals that did not copulate prior to sacrifice. Finally, regardless of experience, animals that had sex prior to sacrifice had significantly more D2-ir cells that expressed Fos than animals that did not copulate (Figure 3C). A heat map depicting the relationship between the fraction of D2-ir cells that expressed Fos and sexual behavior is presented in Figure 3C.

**Figure 4:**
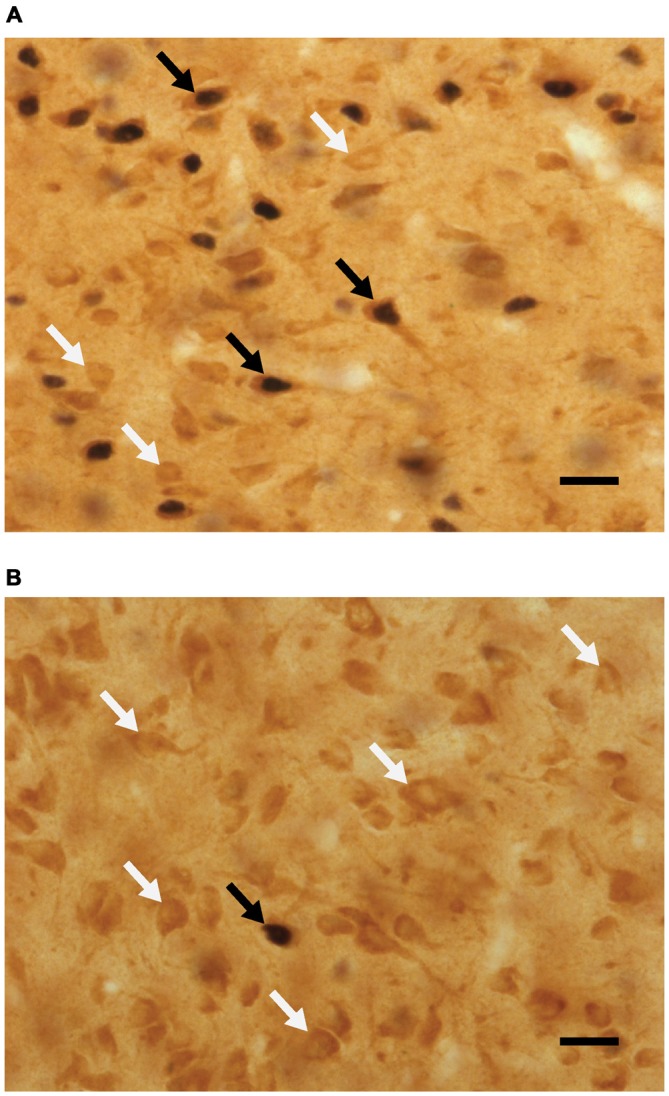
**Micrographs portraying representative-colocalized D2- and Fos-immunoreactive cells (black arrows), together with D2-immunoreactive cells (white arrows) in the mPOA.** Representative micrographs taken from **(A)** a sexually experienced male rat that mated on the day of testing and **(B)** sexually experienced rat that did not mate on the day of testing. Scale bar is 20 μm.

Analyses of percent of Fos-positive cells without D2-like receptors, using a two-way ANOVA, revealed a main effect of sex (*F*_(1,42)_ = 15.795, *p* < 0.001), where the percent of Fos cells not containing D2 was higher in animals that did not copulate before being sacrificed. See Figure 4 for representative micrographs. Following are the percent of Fos-positive cells not containing D2 (mean ± SEM): naive/no-sex, 11.45 ± 1.4; naive/sex, 7.98 ± 0.8; experienced/no-sex, 13.04 ± 1.4; experienced/sex, 6.51 ± 1.0.

In order to verify that changes in immunoreactivity were specific to the mPOA, the number of immuno-reactive cells for Fos and D2 was also examined in the diagonal band of Broca, a region found at the same anteroposterior level as the mPOA. Analyses revealed no significant differences in the number of D2-ir, Fos-ir, or colocalized cells between any of the four groups. Specifically, the number of D2-ir cells was as follows for each group (mean ± SEM): Exp-No Sex, 105 ± 3.08; Exp-Sex, 104 ± 4.62; Naïve-No Sex, 118 ± 5.46; Naïve-Sex, 112 ± 6.01. The number of Fos-ir cells was as follows for each group (mean ± SEM): Exp-No Sex, 1.5 ± 0.7; Exp-Sex, 2.7 ± 0.6; Naïve-No Sex, 1.5 ± 0.58; Naïve-Sex, 3.9 ± 0.6. The number of cells containing both D2 and Fos was as follows for each group (Mean ± SEM): Exp-No Sex, 1.3 ± 0.60; Exp-Sex, 1.85 ± 0.36; Naïve-No Sex, 1.3 ± 0.56; Naïve-Sex, 2.5 ± 0.48.

Behavioral measures are presented here in Table 1. Consistent with previous findings, experienced animals had shorter latencies to mount, intromit, and ejaculate. Sexual experience did not influence mount or intromission frequency.

**Table 1:** **Copulatory behavior**.

Behavior	Experience	Mean ± SEM^1^	*t*-value	*p*-value
Mount latency	*Naïve*	11.26 ± 4.31	−2.57	<0.05
	*Experienced*	0.20 ± 0.09		
Intromission latency	*Naïve*	16.72 ± 4.91	−3.31	<0.01
	*Experienced*	0.47 ± 0.15		
Ejaculation latency	*Naïve*	12.66 ± 1.18	−4.95	<0.001
	*Experienced*	5.33 ± 0.89		
Total mounts	*Naïve*	5.73 ± 1.45	−0.30	ns
	*Experienced*	6.27 ± 1.14		
Total intromissions	*Naïve*	7.55 ± 0.72	0	ns
	*Experienced*	7.55 ± 1.19		

*^1^Mean and SEM for times are reported in minutes*.

## Discussion

Studies show that dopamine agonists microinjected into the mPOA facilitate sexual behavior, whereas microinjections of dopamine antagonists impair copulation, genital reflexes, and sexual motivation (Hull and Dominguez, 2015). Moreover, dopamine levels increase in the mPOA during precopulatory exposure to an estrous female and during copulation (Hull and Dominguez, 2015). Consistent with other studies, we showed that mating increases Fos in the mPOA of male rats (Hull and Dominguez, 2015). We also showed that sexual experience facilitates sexual behavior in males, as measured by decreased latencies to initiate mating and achieve ejaculation. This, too, is consistent with previous studies. We are the first to show that mating increases Fos in D2-containing cells in the mPOA and moreover, that this stimulation is experience dependent. We also found that repeated sexual experience increases the number of cells containing D2-like immunoreactivity. Interestingly, the number of Fos cells not containing D2 was higher in animals that did not copulate before being sacrificed, supporting the importance of dopamine-receptor containing cells for mating, as copulation stimulated mostly D2-containing cells. Lastly, we found correlations between the number of Fos-ir cells and intromissions, D2-ir and mount latency, and percent Fos/D2 and intromissions, as a function of experience.

Our results show that mating stimulated cells containing D2-like receptors in the mPOA. Generally, activation of receptors in the D1 family (D1 and D5) of receptors stimulates adenylyl cyclase, while those in the D2 family (D2, D3, and D4) inhibit the formation of cAMP by inhibiting the enzyme adenylate cyclase (Sibley and Monsma, 1992; Gingrich and Caron, 1993). Consequently, separating specific contributions of D1 vs. D2 in the mPOA to the expression of male sexual behavior becomes important. And while we did not directly examine activation of D1 receptors, we should like to speculate on the significance of our findings on the role of D1/D2 in the mPOA on sexual behavior. The Hull model of dopaminergic influence on sex (Dominguez and Hull, 2005) posits that stimulation of D1 and D2 receptors in the mPOA may have some synergistic and some opposing behavioral effects.

Stimulation of D1 receptors increased the number of ex copula erections but decreased the number of seminal emissions; conversely a D1 antagonist had the opposite effect, decreased erections and increased seminal emissions (Hull et al., 1992). Therefore, stimulation of D1-like receptors may provide the “engine” for erections. In this same study, a low dose of apomorphine increased erections, and this effect was fully blocked by the D1 antagonist SCH-23390 and partially blocked by the D2 antagonist raclopride, suggesting that both receptor types contributed to apomorphine’s effects, but that the D1 receptor was more effective. On the other hand, a high dose of apomorphine increased seminal emissions, and this effect was blocked by the D2 antagonist raclopride and slightly enhanced by the D1 antagonist SCH-23390 (Markowski et al., 1994), suggesting that potent stimulation of D2-like receptors may shift the autonomic balance to favor seminal emission and inhibit erection. Therefore, D1 and D2 receptors in the mPOA have different thresholds of activation and different effects on autonomic control of genital reflexes. Finally, microinjections of THP into the mPOA facilitate copulation (Markowski et al., 1994), whereas a high dose of the D2 agonist quinelorane delayed the start and slowed the rate of copulation while decreasing the number of intromissions required to trigger an ejaculation (Hull et al., 1989). Therefore, synergy between D1 and D2 receptors in the mPOA occurs, in that activation of D2 receptors may be required to disinhibit erections, which are then activated by stimulation of D1 receptors by low to moderate levels of dopamine. In contrast, intense or more prolonged stimulation of D2 receptors may shift the autonomic balance to favor ejaculations. According to this model a high threshold mechanism, activated by stimulation of D2 receptors, facilitates seminal emissions and inhibits erections (viz. sympathetic activation); if true, then our results are in line with this model. These are also consistent with other animal models, such as the Japanese quail, where D1 and D2 manipulations have differential effects on appetitive and consummatory behavior (Balthazart et al., 1997). Ejaculations in our study were associated with increased stimulation of D2-containing cells, as evidenced by the presence of Fos in D2-containing cells. This is, again, consistent with the Hull model, in which stronger dopaminergic stimulation of D2 receptor-containing cells precedes ejaculations. Our results also showed that sexually inexperienced animals had greater activation of D2-containing cells, suggesting that these animals required both stimulation of D2-like receptors to facilitate disinhibition of genital reflexes and also required greater stimulation of D2-containg cells to achieve ejaculations. This too is consistent with the Hull model.

The short and long form of the D2 receptor vary in size and function (Moreira et al., 2010). Here we examined the D2 long form, which functions primarily postsynaptically. However, we did not examine the function of D1 receptors. Nevertheless, colocalization of D2 receptors with Fos suggests some involvement of D1 receptors since this activation would presumably require D1 stimulation. Stimulants such as cocaine, d-amphetamine, and methamphetamine produce large increases in striatal Fos levels (Graybiel et al., 1990; Carney et al., 1991; Young et al., 1991), signifying that increased dopamine increases c-fos expression. This increase, however, was blocked by selective D1-receptor antagonists (Young et al., 1991). Other studies, however, do show interactive effects of D1 and D2 receptors on Fos expression, as in the lateral habenula (Wirtshafter and Krebs, 1997). Wirtshafter and Krebs injected rats with various doses of the selective D2 agonist quinpirole either alone or in combination with various doses of the selective D1 agonist A-77636. Individually, the selective agonists induced small increases in Fos-ir, but combinations of the two drugs resulted in a robust increase (Wirtshafter and Krebs, 1997), indicating an important synergism between D1 and D2 receptors (Gerfen et al., 1995; Keefe and Gerfen, 1995). Since increased c-Fos expression is associated with depolarization, the upregulation of c-Fos in D2-containing cells after mating, as shown here, backs the idea that D1 and D2 in the mPOA have synergistic influences on copulation. Namely, the initiation of mating stimulates the D1 receptor through low to moderate levels of dopamine, which is then followed by intense or more prolonged stimulation of D2 receptors, shifting the autonomic balance to favor ejaculations.

Regarding sexual experience, we show that mating stimulates cells containing putative D2 receptors in the mPOA and that this stimulation is greater in the mPOA of previously inexperienced males undergoing their first sexual encounter. This finding is noteworthy because sexually experienced animals display increased sexual efficiency, as evidenced by an increase in ejaculation frequency and decreased latencies, when compared to inexperienced males (Hull and Dominguez, 2015). This difference in activation of D2 may play a role in the behavioral differences that endure between experienced and inexperienced animals. Specifically, one can speculate that dopamine-sensitive cells require less stimulation with repeated and prolonged experience, as evidenced by lower colocalization in the experienced vs. inexperienced animals in our study. Finally, we also discovered a greater number of putative D2-ir cells in experienced animals. This change correlated with sexual behaviors. Sexually experienced animals required less time to initiate mating, as evidence by mount latency. This latency negatively correlated with the number of D2-ir cells, meaning that the more time required before mating, the lower the number of D2-ir. This suggests that sexual experience increased levels of D2, which is associated with decreased latency to mate. Other experience-dependent behavioral measures correlated with Fos-ir. The number of Fos-ir cells positively correlated with number of intromissions in inexperienced animals, suggesting that sexually inexperienced animals required greater stimulation of the mPOA to achieve ejaculations, as these animals mated to only one ejaculation. Conversely, the repeated stimulation resulting from more intromissions may have led to greater activity in the mPOA. Finally, The number of D2-cells containing Fos positively correlated with the number of intromissions, again in sexually inexperienced animals. As with Fos, this might have a two-pronged explanation. Namely, sexually inexperienced animals required greater stimulation of the D2-containing cells to achieve ejaculations, and conversely the prolonged exposure to dopamine resulting from greater intromissions may have led to greater activity in the mPOA.

In conclusion, we showed that sexual experience facilitated sexual behavior and that these changes had behavioral correlates in the mPOA. Namely, cells containing D2-like dopamine receptors colocalized with Fos. This colocalization was highest in animals mating for the first time. Sexually experienced animals also had more D2-like dopamine receptors in their mPOA. These neural correlates and associated neuroplasticity may account, at least in part, for the behavioral changes that follow sexual experience in male rats.

## Author Contributions

VLN, RGW performed behavioral and histological experiments, and assisted writing the manuscript. CLR performed Western immunoblots and assisted with in writing the manuscript. JRM and DJT assisted with histological and behavioral experiments. JMD designed experiments and wrote the manuscript.

## Funding

This research was supported by startup funds from The University of Texas at Austin, College of Liberal Arts to JMD and by NIH grant R01-DA032789 to JMD.

## Conflict of Interest Statement

The authors declare that the research was conducted in the absence of any commercial or financial relationships that could be construed as a potential conflict of interest.

## References

[B1] Balthazart J. Castagna C. Ball G. F. (1997). Differential effects of D1 and D2 dopamine-receptor agonists and antagonists on appetitive and consummatory aspects of male sexual behavior in Japanese quail. Physiol. Behav. 62, 571–580. 10.1016/s0031-9384(97)00163-7 9272666

[B2] Baum M. J. Everitt B. J. (1992). Increased expression of c-fos in the medial preoptic area after mating in male rats: role of afferent inputs from the medial amygdala and midbrain central tegmental field. Neuroscience 50, 627–646. 10.1016/0306-4522(92)90452-8 1436507

[B3] Bitran D. Hull E. M. Holmes G. M. Lookingland K. J. (1988). Regulation of male rat copulatory behavior by preoptic incertohypothalamic dopamine neurons. Brain Res. Bull. 20, 323–331. 10.1016/0361-9230(88)90062-7 3130153

[B4] Björklund A. Lindvall O. Nobin A. (1975). Evidence of an incerto-hypothalamic dopamine neurone system in the rat. Brain Res. 89, 29–42. 10.1016/0006-8993(75)90131-6 238718

[B5] Boundy V. A. Luedtke R. R. Artymyshyn R. P. Filtz T. M. Molinoff P. B. (1993). Development of polyclonal anti-D2 dopamine receptor antibodies using sequence-specific peptides. Mol. Pharmacol. 43, 666–676. 10.1111/j.1471-4159.1993.tb03504.x 8502224

[B6] Bressler S. C. Baum M. J. (1996). Sex comparison of neuronal Fos immunoreactivity in the rat vomeronasal projection circuit after chemosensory stimulation. Neuroscience 71, 1063–1072. 10.1016/0306-4522(95)00493-9 8684610

[B7] Carney J. M. Tolliver B. Carney J. P. Kindy M. S. (1991). Selective effects of behaviorally active doses of methamphetamine on mRNA expression in the gerbil brain. Neuropharmacology 30, 1011–1019. 10.1016/0028-3908(91)90114-q 1922692

[B8] Da Prada M. Carruba M. Saner A. O’Brien A. Pletscher A. (1973). The action L-DOPA on sexual behaviour of male rats. Brain Res. 55, 383–389. 10.1016/0006-8993(73)90303-x 4541349

[B9] Dominguez J. M. (2009). A role for preoptic glutamate in the regulation of male reproductive behavior. Neuroscientist 15, 11–19. 10.1177/1073858408322679 19218227

[B10] Dominguez J. M. Gil M. Hull E. M. (2006). Preoptic glutamate facilitates male sexual behavior. J. Neurosci. 26, 1699–1703. 10.1523/JNEUROSCI.4176-05.2006 16467517 PMC6793616

[B11] Dominguez J. M. Hull E. M. (2005). Dopamine, the medial preoptic area and male sexual behavior. Physiol. Behav. 86, 356–368. 10.1016/j.physbeh.2005.08.006 16135375

[B12] Farooqui S. M. Prasad C. Ali M. (1992). Production and characterization of a monoclonal antibody to dopamine D2 receptor: comparison with a polyclonal antibody to a different epitope. Biochem. Biophys. Res. Commun. 184, 661–667. 10.1016/0006-291x(92)90640-7 1349476

[B13] Ferrari F. Ottani A. Giuliani D. (2002). Influence of sildenafil on central dopamine-mediated behaviour in male rats. Life Sci. 70, 1501–1508. 10.1016/s0024-3205(01)01515-6 11895101

[B14] Gerfen C. R. Keefe K. A. Gauda E. B. (1995). D1 and D2 dopamine receptor function in the striatum: coactivation of D1- and D2-dopamine receptors on separate populations of neurons results in potentiated immediate early gene response in D1-containing neurons. J. Neurosci. 15, 8167–8176. 8613751 10.1523/JNEUROSCI.15-12-08167.1995 PMC6577952

[B15] Gil M. Bhatt R. Picotte K. B. Hull E. M. (2011). Oxytocin in the medial preoptic area facilitates male sexual behavior in the rat. Horm. Behav. 59, 435–443. 10.1016/j.yhbeh.2010.12.012 21195714 PMC3081415

[B16] Gingrich J. A. Caron M. G. (1993). Recent advances in the molecular biology of dopamine receptors. Annu. Rev. Neurosci. 16, 299–321. 10.1146/annurev.neuro.16.1.299 8460895

[B17] Giuliano F. Bernabé J. Brown K. Droupy S. Benoit G. Rampin O. (1997). Erectile response to hypothalamic stimulation in rats: Role of peripheral nerves. Am. J. Physiol. 273, R1990–R1997. 9435653 10.1152/ajpregu.1997.273.6.R1990

[B18] Graybiel A. M. Moratalla R. Robertson H. A. (1990). Amphetamine and cocaine induce drug-specific activation of the c-fos gene in striosome-matrix compartments and limbic subdivisions of the striatum. Proc. Natl. Acad. Sci. U S A 87, 6912–6916. 10.1073/pnas.87.17.6912 2118661 PMC54648

[B19] Hull E. M. Bitran D. Pehek E. A. Warner R. K. Band L. C. (1986). Dopaminergic control of male sex behavior in rats: effects of an intracerebrally infused agonist. Brain Res. 370, 73–81. 10.1016/0006-8993(86)91106-6 3011196

[B20] Hull E. M. Dominguez J. M. (2006). Getting his act together: roles of glutamate, nitric oxide and dopamine in the medial preoptic area. Brain Res. 1126, 66–75. 10.1016/j.brainres.2006.08.031 16963001

[B21] Hull E. M. Dominguez J. M. (2015). “Male sexual behavior,” in Knobil and Neill’s Physiology of Reproduction, 4th Edn, eds Plant T. M. Zeleznik A. J. (New York, NY: Elsevier), 2211–2285.

[B22] Hull E. M. Eaton R. C. Markowski V. P. Moses J. Lumley L. A. Loucks J. A. (1992). Opposite influence of medial preoptic Dl and D2 receptors on genital reflexes: implications for copulation. Life Sci. 51, 1705–1713. 10.1016/0024-3205(92)90299-5 1359367

[B23] Hull E. M. Lumley L. A. Matuszewich L. Dominguez J. Moses J. Lorrain D. S. (1994). The roles of nitric oxide in sexual function of male rats. Neuropharmacology 33, 1499–1504. 10.1016/0028-3908(94)90054-x 7532834

[B24] Hull E. M. Warner R. K. Bazzett T. J. Eaton R. C. Thompson J. T. Scaletta L. L. (1989). D2/D1 ratio in the medial preoptic area affects copulation of male rats. J. Pharmacol. Exp. Ther. 251, 422–427. 2572689

[B25] Keefe K. A. Gerfen C. R. (1995). D1–D2 dopamine receptor synergy in striatum: effects of intrastriatal infusions of dopamine agonists and antagonists on immediate early gene expression. Neuroscience 66, 903–913. 10.1016/0306-4522(95)00024-d 7651617

[B26] Kleitz-Nelson H. K. Dominguez J. M. Ball G. F. (2010a). Dopamine release in the medial preoptic area is related to hormonal action and sexual motivation. Behav. Neurosci. 124, 773–779. 10.1037/a0021490 21133533 PMC3003599

[B27] Kleitz-Nelson H. K. Dominguez J. M. Cornil C. A. Ball G. F. (2010b). Is sexual motivational state linked to dopamine release in the medial preoptic area? Behav. Neurosci. 124, 300–304. 10.1037/a0018767 20364890 PMC2852173

[B29] Lidow M. S. Goldman-Rakic P. S. Rakic P. Innis R. B. (1989). Dopamine D2 receptors in the cerebral cortex: distribution and pharmacological characterization with [3H]raclopride. Proc. Natl. Acad. Sci. U S A 86, 6412–6416. 10.1073/pnas.86.16.6412 2548214 PMC297850

[B30] Malsbury C. W. (1971). Facilitation of male rat copulatory behavior by electrical stimulation of the medial preoptic area. Physiol. Behav. 7, 797–805. 10.1016/0031-9384(71)90042-4 5134017

[B31] Markowski V. P. Eaton R. C. Lumley L. A. Moses J. Hull E. M. (1994). A D1 agonist in the MPOA area facilitates copulation of male rats. Pharmacol. Biochem. Behav. 47, 483–486. 10.1016/0091-3057(94)90147-3 7911575

[B32] Marson L. McKenna K. E. (1994). Serotonergic neurotoxic lesions facilitate male sexual reflexes. Pharmacol. Biochem. Behav. 47, 883–888. 10.1016/0091-3057(94)90292-5 7518085

[B33] McHenry J. A. Bell G. A. Parrish B. P. Hull E. M. (2012). Dopamine D1 receptors and phosphorylation of dopamine- and cyclic AMP-regulated phosphoprotein-32 in the medial preoptic area are involved in experience-induced enhancement of male sexual behavior in rats. Behav. Neurosci. 126, 523–529. 10.1037/a0028707 22708956 PMC3409344

[B34] Meador-Woodruff J. H. Mansour A. Bunzow J. R. Van Tol H. H. Watson S. J. Jr. Civelli O. (1989). Distribution of D2 dopamine receptor mRNA in rat brain. Proc. Natl. Acad. Sci. U S A 86, 7625–7628. 10.1073/pnas.86.19.7625 2529545 PMC298119

[B35] Mendelson S. D. Pfaus J. G. (1989). Level searching: a new assay of sexual motivation in the male rat. Physiol Behav 45, 337–341. 10.1016/0031-9384(89)90136-4 2756020

[B36] Miller S. M. Lonstein J. S. (2009). Dopaminergic projections to the medial preoptic area of postpartum rats. Neuroscience 159, 1384–1396. 10.1016/j.neuroscience.2009.01.060 19409227 PMC2888488

[B37] Moore K. E. Lookingland K. J. (1995). “Dopaminergic neuronal systems in the hypothalamus,” in Psychopharmacology: The Fourth Generation of Progress, eds Bloom F. E. Kupfer D. J. (New York, NY: Raven Press), 245–246.

[B38] Moreira I. S. Shi L. Freyberg Z. Ericksen S. S. Weinstein H. Javitch J. A. (2010). “Structural basis of dopamine receptor activation,” in The Dopamine Receptors, 2nd Edn, ed. Neve K. A. (New York, NY: Humana Press), 47–74.

[B39] Nutsch V. L. Will R. G. Hattori T. Tobiansky D. J. Dominguez J. M. (2014). Sexual experience influences mating-induced activity in nitric oxide synthase-containing neurons in the medial preoptic area. Neurosci. Lett. 579, 92–96. 10.1016/j.neulet.2014.07.021 25058433

[B40] Oomura Y. Aou S. Koyama Y. Yoshimatsu H. (1988). Central control of sexual behavior. Brain Res. Bull. 20, 863–870. 10.1016/0361-9230(88)90103-7 3409059

[B41] Paglietti E. Quarantotti B. P. Mereu G. Gessa G. L. (1978). Apomorphine and L-DOPA lower ejaculation threshold in the male rat. Physiol. Behav. 20, 559–562. 10.1016/0031-9384(78)90247-0 684090

[B42] Pehek E. A. Thompson J. T. Eaton R. C. Bazzett T. J. Hull E. M. (1988a). Apomorphine and haloperidol, but not domperidone, affect penile reflexes in rats. Pharmacol. Biochem. Behav. 31, 201–208. 10.1016/0091-3057(88)90334-6 3252251

[B44] Pehek E. A. Warner R. K. Bazzett T. J. Bitran D. Band L. C. Eaton R. C. (1988b). Microinjection of cis-flupenthixol, a dopamine antagonist, into the medial preoptic area impairs sexual behavior of male rats. Brain Res. 443, 70–76. 10.1016/0006-8993(88)91599-5 3359281

[B43] Pehek E. A. Thompson J. T. Hull E. M. (1989). The effects of intracranial administration of the dopamine agonist apomorphine on penile reflexes and seminal emission in the rat. Brain Res. 500, 325–332. 10.1016/0006-8993(89)90328-4 2605500

[B45] Pfaus J. G. Heeb M. M. (1997). Implications of immediate-early gene induction in the brain following sexual stimulation of female and male rodents. Brain Res. Bull. 44, 397–407. 10.1016/s0361-9230(97)00219-0 9370204

[B46] Pfaus J. G. Phillips A. G. (1989). Differential effects of dopamine receptor antagonists on the sexual behavior of male rats. Psychopharmacology (Berl.) 98, 363–368. 10.1007/bf00451688 2568656

[B47] Pfaus J. G. Phillips A. G. (1991). Role of dopamine in anticipatory and consummatory aspects of sexual behavior in the male rat. Behav. Neurosci. 105, 727–743. 10.1037/0735-7044.105.5.727 1840012

[B48] Rampin O. Jérôme N. Suaudeau C. (2003). Proerectile effects of apomorphine in mice. Life Sci. 72, 2329–2336. 10.1016/s0024-3205(03)00122-x 12639699

[B49] Robertson G. S. Pfaus J. G. Atkinson L. J. Matsumura H. Phillips A. G. Fibiger H. C. (1991). Sexual behavior increases c-fos expression in the forebrain of the male rat. Brain Res. 564, 352–357. 10.1016/0006-8993(91)91477-i 1810635

[B50] Rodríguez-Manzo G. Pellicer F. Larsson K. Fernández-Guasti A. (2000). Stimulation of the medial preoptic area facilitates sexual behavior but does not reverse sexual satiation. Behav. Neurosci. 114, 553–560. 10.1037/0735-7044.114.3.553 10883805

[B51] Sakata M. Farooqui S. M. Prasad C. (1992). Post-transcriptional regulation of loss of rat striatal D2 dopamine receptor during aging. Brain Res. 575, 309–314. 10.1016/0006-8993(92)90095-q 1533340

[B52] Shimura T. Yamamoto T. Shimokochi M. (1994). The medial preoptic area is involved in both sexual arousal and performance in male rats: re-evaluation of neuron activity in freely moving animals. Brain Res. 640, 215–222. 10.1016/0006-8993(94)91875-9 8004448

[B53] Sibley D. R. Monsma F. J. Jr. (1992). Molecular biology of dopamine receptors. Trends Pharmacol. Sci. 13, 61–69. 10.1016/0165-6147(92)90025-2 1561715

[B54] Simerly R. B. Swanson L. W. (1986). The organization of neural inputs to the medial preoptic nucleus of the rat. J. Comp. Neurol. 246, 312–342. 10.1002/cne.902460304 3517086

[B55] Simerly R. B. Swanson L. W. (1988). Projections of the medial preoptic nucleus: a *Phaseolis vulgaris* leucoagglutinin anterograde tract-tracing study in the rat. J. Comp. Neurol. 270, 209–242. 10.1002/cne.902700205 3259955

[B56] Swaney W. T. Dubose B. N. Curley J. P. Champagne F. A. (2012). Sexual experience affects reproductive behavior and preoptic androgen receptors in male mice. Horm. Behav. 61, 472–478. 10.1016/j.yhbeh.2012.01.001 22266118 PMC3319191

[B57] Swanson L. W. (2004). Brain Maps: Structure of the Rat Brain. San Diego, CA: Academic Press.

[B58] Tobiansky D. J. Hattori T. Scott J. M. Nutsch V. L. Roma P. G. Dominguez J. M. (2012). Mating-relevant olfactory stimuli activate the rat brain in an age-dependent manner. Neuroreport 23, 1077–1083. 10.1097/wnr.0b013e32835b6ec1 23147712

[B59] Veening J. G. Coolen L. M. (1998). Neural activation following sexual behavior in the male and female rat brain. Behav. Brain Res. 92, 181–193. 10.1016/s0166-4328(97)00190-3 9638960

[B60] Will R. G. Nutsch V. L. Turner J. M. Hattori T. Tobiansky D. J. Dominguez J. M. (2015). Astrocytes in the medial preoptic area modulate ejaculation latency in an experience-dependent fashion. Behav. Neurosci. 129, 68–73. 10.1037/bne0000026 25621794 PMC6822610

[B61] Wirtshafter D. Krebs J. C. (1997). Interactive effects of stimulation of D1 and D2 dopamine receptors on Fos expression in the lateral habenula. Brain Res. 750, 245–250. 10.1016/s0006-8993(96)01353-4 9098550

[B62] Young S. T. Porrino L. J. Iadarola M. J. (1991). Cocaine induces striatal c-fos-immunoreactive proteins via dopaminergic D1 receptors. Proc. Natl. Acad. Sci. U S A 88, 1291–1295. 10.1073/pnas.88.4.1291 1825356 PMC51003

